# Discrimination of fearful and happy body postures in 8-month-old infants: an event-related potential study

**DOI:** 10.3389/fnhum.2014.00531

**Published:** 2014-07-24

**Authors:** Manuela Missana, Purva Rajhans, Anthony P. Atkinson, Tobias Grossmann

**Affiliations:** ^1^Early Social Development Group, Max Planck Institute for Human Cognitive and Brain SciencesLeipzig, Germany; ^2^Department of Psychology, Durham UniversityDurham, UK

**Keywords:** emotion, infants, body expressions, ERP, development

## Abstract

Responding to others’ emotional body expressions is an essential social skill in humans. Adults readily detect emotions from body postures, but it is unclear whether infants are sensitive to emotional body postures. We examined 8-month-old infants’ brain responses to emotional body postures by measuring event-related potentials (ERPs) to happy and fearful bodies. Our results revealed two emotion-sensitive ERP components: body postures evoked an early N290 at occipital electrodes and a later Nc at fronto-central electrodes that were enhanced in response to fearful (relative to happy) expressions. These findings demonstrate that: (a) 8-month-old infants discriminate between static emotional body postures; and (b) similar to infant emotional face perception, the sensitivity to emotional body postures is reflected in early perceptual (N290) and later attentional (Nc) neural processes. This provides evidence for an early developmental emergence of the neural processes involved in the discrimination of emotional body postures.

## Introduction

Reading others’ emotional expressions is a vital skill that helps us predict others’ actions and guide our own behavior during social interactions (Frith, 2009). Emotional communication is inherently multidimensional and multisensory in nature as emotional information can be gleaned from various sources such as the face, the voice, the body posture and motion of a person (Heberlein and Atkinson, 2009). The bulk of research investigating emotion expression perception has focused on facial and vocal expressions (Belin et al., 2012). Much less work has been dedicated to understanding the perception of emotional body expressions, even though body expressions may be the most evolutionarily preserved and immediate means of conveying emotional information (de Gelder, 2006). The work on emotional body expressions has revealed that adults are readily able to detect and recognize various emotions from body expressions (de Gelder, 2009; Atkinson, 2013) and that in some instances emotional body cues can even be detected in the absence of conscious awareness (see Tamietto and de Gelder, 2010). Furthermore, there is recent evidence to show relatively better discrimination between positive and negative emotions from body cues when compared to facial cues (Aviezer et al., 2012). This ability to recognize emotions from body expressions relies on specific brain processes localized principally in the right hemisphere, including superior temporal, somatosensory and premotor cortices (Heberlein et al., 2004; Heberlein and Saxe, 2005; de Gelder, 2006; Grèzes et al., 2007; Atkinson, 2013). Concerning the temporal dynamics of the brain processes involved in differential responding to emotional body expressions in adults, using event-related brain potentials (ERPs), it has been shown that fearful body postures evoke enhanced activity during early stages of visual processing (van Heijnsbergen et al., 2007) and further result in sustained activity over fronto-central brain regions during later processing stages (Stekelenburg and de Gelder, 2004).

Only recently, research has begun to examine how the ability to perceive and respond to others’ emotional body expressions develops during infancy. Specifically, Zieber et al. (2014) examined infants’ sensitivity to emotional body expressions in a series of behavioral experiments with 6.5-month-old infants (using video full-light body expressions taken from Atkinson et al., 2004, 2007). In this study, 6.5-month-olds showed a visual preference for happy over neutral body expressions and were shown to look longer at body-voice pairings that conveyed congruent emotional information (happiness or anger) than incongruent emotional information. Critically, these effects were specific to body expressions presented in an upright orientation, since infants did not show any difference in their looking responses when the body expression was presented upside-down (Zieber et al., 2014). While these findings provide first insights into infants’ perceptual sensitivity to emotional body expressions, a number of vital remaining issues were addressed in a recent ERP study (Missana et al., 2014a).

In this study Missana et al. (2014a) investigated the developmental emergence of infants’ neural sensitivity to emotional body expressions by presenting 4- and 8-month-olds with upright and inverted happy and fearful dynamic body expressions using point-light displays (PLDs). This ERP study yielded three main findings with respect to infants’ developing sensitivity to emotional body expressions. First, similar to prior work using facial and vocal emotional expressions (Nelson and de Haan, 1996; Grossmann et al., 2005; Peltola et al., 2009) 8-month-olds, but not 4-month-olds, showed a neural discrimination between fearful and happy body movements, suggesting that the ability to process the emotional content of body movements develops during the first year of life. Second, in line with prior work that has shown that body expression perception in adults and infants is impaired by stimulus inversion (Atkinson, 2013; Zieber et al., 2014), the differential ERP responses to fearful vs. happy expressions in 8-month-old infants was mainly evident in the upright condition but not in the inverted condition. Third, in agreement with previous findings of right-hemisphere lateralization of emotional body-expression processing in adults (Heberlein et al., 2004; Heberlein and Saxe, 2005; Grèzes et al., 2007), 8-month-old infants emotion-sensitive brain responses were lateralized to the right hemisphere.

Given what has been shown in recent work (Missana et al., 2014a; Zieber et al., 2014), another important question that arises is, once infants are sensitive to body expressions, how flexible are they in detecting them? Specifically, can infants discriminate between emotional body expressions in the absence of motion cues like adults can (Atkinson et al., 2004; de Gelder et al., 2004; Stekelenburg and de Gelder, 2004; Atkinson et al., 2012)? Such an extension of prior work is critical because (a) it provides a developmental perspective on emotional body expression processing by allowing for a comparison between prior ERP findings with adults (Stekelenburg and de Gelder, 2004; van Heijnsbergen et al., 2007) and the current infant data; and (b) it establishes a link to the literature on facial expression processing since most prior ERP work on infants’ processing of emotional facial expressions has been focused on static but not dynamic facial expressions (Missana et al., 2014b).

In order to examine the question posed above, we conducted an experiment in which we presented a group of 8-month-old infants with static photographs of upright and inverted happy and fearful body expressions while measuring their ERPs. We hypothesized that if 8-month-olds are sensitive to emotional information conveyed through the body even in the absence of motion cues, then they would show evidence for discriminating between fearful and happy emotional body postures in their ERP responses. Regarding this hypothesis it is important to emphasize that from prior work with adults (Atkinson et al., 2004, 2007) we know that while inversion of body expressions impairs the recognition of emotion from body expressions, it does not completely abolish it. That is, adults’ emotion recognition rates from inverted body expressions are still above chance. Similarly, in prior ERP work with infants (Missana et al., 2014a) using dynamic body expressions, while there was a significant main effect of emotion (fearful, happy), no evidence for an interaction between the emotion and orientation (upright, inverted) was obtained, which is probably to do with the fact that ERP modulations occurred in a similar direction for upright and inverted displays of emotion. However, further analysis revealed that only in the upright condition did infants’ neural responses discriminate between emotions (Missana et al., 2014a). We hypothesized that neural evidence for emotion discrimination is related to the orientation of the body; however, given previous findings, we expected that this effect might not be directly reflected in a significant interaction between stimulus orientation and emotion. This hypothesis is also based on prior work that has shown that body expression perception in adults and in infants is impaired (but not completely abolished) by stimulus inversion (Stekelenburg and de Gelder, 2004; Atkinson et al., 2007; Missana et al., 2014a; Zieber et al., 2014). We therefore predicted that infants’ discrimination between emotions would mainly be evident in the upright condition but not, or at least not as clearly, in the inverted condition. More specifically, we focused our investigation on infant ERP components linked to early visual processes (N290) at posterior sites, and later attentional processes (Nc) at anterior sites, which are reliability observed in response to visual stimuli and known to be modulated by emotional information (Grossmann et al., 2006; Kobiella et al., 2008; Peltola et al., 2009). Based on prior work (Missana et al., 2014a), we had also planned to assess effects on a late ERP component referred to as the Pc, evoked at temporal and parietal electrodes, that has been found to be modulated by dynamic emotional body expressions. However, already at the level of the visual inspection of the ERP data there was no discernible Pc observed in the ERPs, which prevented us from studying this component further. This approach also allowed us (a) to assess potential differences between statically (current study) and dynamically presented body expressions (Missana et al., 2014a); and (b) to examine whether the body inversion effects and hemispheric lateralization of the ERP responses observed in prior work using PLDs (Missana et al., 2014a) could be replicated with a different group of 8-month-infants using static body expressions.

## Methods and materials

### Participants

The final sample consisted of 15 8-month-old infants aged between 243 and 261 days (10 females, *Median age* = 251, *Range* = 18 days). An additional 23 8-month-old infants were tested, but were excluded from the final sample due to fussiness (*n* = 5), too many artifacts (*n* = 16) and experimenter error (*n* =2). Note that an attrition rate at this level is within the normal range for an infant ERP study (DeBoer et al., 2005). The infants were born full-term (between 37 and 41 weeks) and had a normal birth weight (>2500 g). Ethical approval was obtained from the ethics committee of the Medical School at the University of Leipzig. All parents gave written informed consent prior to the study and were paid for their children’s participation. The children were given a toy after the session.

### Stimuli

**Figure 1:**
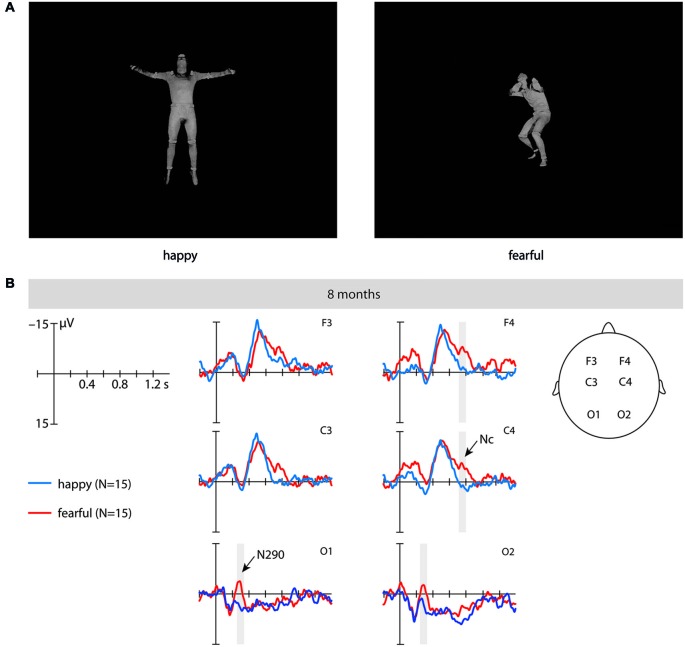
**This figure shows examples of the stimuli and the event-related brain potentials (ERPs)**. **(A)** These are examples of the static full-light body expression stimuli (upright) used in the study. **(B)** This shows the ERPs at fronto-central and occipital electrodes time-locked to the stimulus onset in 8-month-old infants elicited by fearful upright (red) and happy upright (blue) static full-light body expressions. The time windows during which significant differences between fearful and happy body expressions were observed are marked in gray. Note that negativity is plotted upward.

The stimulus material consisted of full-light static body expressions displaying six different fearful and six different happy expressions (from Atkinson et al., 2004). These expressions were taken from the same actors posing the same emotions as in a previous ERPs study (Missana et al., 2014a) by selecting still frames of the full-light version of the body expression recording at the peak of the expression (see Figure 1A). From the original set of eight stimuli per condition used in Missana et al.’s (2014a) study, six stimuli for each emotion were selected on the basis of their recognition rate by a group of adults (Atkinson et al., 2004) (at least 40% mean percentage correct identification of the emotion displayed; chance level was 16.7%). The stimuli had a mean height of 11.9 cm subtending a visual angle of 9.74° (SD = 3.5 cm) and a mean width of 6.5 cm subtending a visual angle of 5.4° (SD = 3.1 cm).

### Procedure

The infants were seated on their parent’s lap in a dimly lit, sound-attenuated and electrically shielded room during testing. In order to rule out that the parents influenced the infants’ responses to the stimuli we asked the parents not to talk or interact with their infant during the course of the experiment (Kobiella et al., 2008). Furthermore, we instructed the parents to look down at the infant but not at the screen and the sessions were video-recorded so that trials during which the parent interacted with the infant could be excluded from the analysis. The stimuli were presented in the center of the screen on a black background, using a 70-Hz, 17-inch computer screen at a distance of 70 cm. Each trial began with an alerting sound and a fixation cross (1000 ms), in order to attract the infants’ attention to the screen, followed by a black screen (400 ms), followed by the full-light static expression (2000 ms). During the inter-trial interval infants were presented with an abstract screensaver for the purpose of keeping infants’ attention. The inter-trial interval lasted at least 1000 ms and varied depending on the infants’ attentiveness, as stimulus presentation was controlled by an experimenter such that stimuli were only presented when infants were looking at the screen. The stimuli were presented in a randomized order with the exception that no two stimuli with the same emotion and orientation combination were presented consecutively. In addition, the sessions were video-recorded to allow for off-line coding of infants’ attention to the screen. The EEG session ended when the infant became fussy, or inattentive.

### ERP analysis

The EEG was recorded from 27 Ag/AgCl electrodes attached to an elastic cap (EasyCap GmbH, Germany) using the 10–20 system of electrode placement. The data was online referenced to the CZ electrode and offline re-referenced to the algebraic mean of the left and right mastoid electrodes. The horizontal electrooculogram (EOG) was recorded from two electrodes (F9, F10) that are part of the cap located at the outer canthi of both eyes. The vertical EOG was recorded from an electrode on the supraorbital ridge (Fp2) that is part of the cap and an additional single electrode on the infraorbital ridge of the right eye. The EEG was amplified using a Porti-32/M-REFA amplifier (Twente Medical Systems International) and digitized at a rate of 500 Hz. Electrode impedances were kept between 5 and 20 kΩ. Data processing for ERP analysis was performed using an in-house software package EEP, commercially available under the name EEProbeTM (Advanced Neuro Technology, Enschede). The raw EEG data was bandpass filtered between 0.3 and 20 Hz. The recordings were segmented into epochs time-locked to the stimulus onset, lasting from 200 ms before onset until the offset of the frame (total duration 2200 ms). The epochs were baseline corrected by subtracting the average voltage in the 200 ms baseline period (prior to video or picture onset) from each post-stimulus data point. The baseline period contained a 200 ms black screen. Data epochs were rejected off-line whenever the standard deviation within a gliding window of 200 ms exceeded 80 μV in any of the two bipolar EOG channels and 60 μV at EEG electrodes. EEG data was also visually inspected offline for artifacts. At each electrode, artifact-free epochs were averaged separately for fearful upright, happy upright, fearful inverted and happy inverted body expressions to compute the ERPs. The mean number of trials presented within each condition was 17.18. The mean number of trials included in the ERP average was 6.60 (*SE* = 0.73) for the fearful upright condition, 7.80 (*SE* = 1.15) for the happy upright condition, 5.93 (*SE* = 0.53) for the fearful inverted condition and 7.60 (*SE* = 1.05) for the happy inverted condition. The criterion for inclusion was a minimum of four trials per condition. The mean number of trials is somewhat lower than in prior infant ERP studies but this is likely to do with the fact that the current design consisted of four conditions, which is more than in prior studies. We note that the low trial number can be seen as a limitation of the current study but want to stress that: (a) in order to allow for comparisons, our procedure and analysis were closely matched to previous work that used dynamic displays of emotional body expressions (Missana et al., 2014a); and (b) conservative rejection criteria were applied so that only artifact-free trials were included in the analysis. In this context it is also important to mention that the Nc, as one of the main components examined in this study, is a rather large deflection, discernible and present in individual infants even when a relatively small number of trials is used (see Hoehl and Wahl, 2012, recent methods paper that provides extensive information regarding ERP measurement and analysis standards for infants and present data from individual infants demonstrating that five trials are sufficient to evoke clear Nc responses). Based on prior ERP work (Leppänen et al., 2007; Kobiella et al., 2008; Peltola et al., 2009; Missana et al., 2014a) and the visual inspection of the ERP data three ERP components distinct in timing (early and late) and topography (occipital and fronto-central) were analyzed. First, to assess ERP effects on the N290 over visual cortex at occipital electrodes (O1, O2), ERPs were statistically analyzed during an early time window of 250–350 ms after stimulus onset. Second, to assess ERP effects on the Nc over frontal cortex at frontal and central electrodes (F3, F4, C3, C4), ERPs were statistically analyzed during a late time window of 700 to 800 ms after stimulus onset. Note that the onset and exact timing of the Nc has been shown to vary considerably across studies and might depend on the stimulus duration and other characteristics of the presentation protocol (de Haan et al., 2003; Grossmann et al., 2007; Leppänen et al., 2007; Luyster et al., 2014; Missana et al., 2014b). Mean amplitude ERP effects for these regions and time windows were assessed in repeated measures ANOVAs with the within-subject factors emotion (happy vs. fear), orientation (upright vs. inverted), and hemisphere (left vs. right). As prior work indicates that emotion-sensitive ERP response were lateralized to the right hemisphere and specific to the upright orientation (Missana et al., 2014a), in addition to the repeated measures ANOVAs, we conducted paired samples *t-*tests to evaluate the orientation specificity and lateralization of the ERP responses.

## Results

*N290*. Our analysis revealed a significant main effect of emotion at occipital electrodes from 250 to 350 ms, *F*_(1, 14)_ = 7.02, *p* = 0.019, *η*^2^ = 0.334, where fearful body expressions (*M* = −1.58 μV, *SE* = 2.59) elicited a more negative N290 than happy body expressions (*M* = 6.14 μV, *SE* = 3.34). Further analysis showed that this effect of emotion was driven by differences in the upright orientation, as upright fearful body expressions (*M* = −2.23 μV, *SE* = 3.04) elicited ERPs that were significantly more negative in their mean amplitude than ERPs elicited by upright happy body expressions (*M* = 6.42 μV, *SE* = 3.42) during this time window, *t*_(14)_ = 2.22, *p* = 0.043 (uncorrected; see Figure 1B and Table 1), whereas no significant differences between emotions were observed for this component when the stimuli were inverted, *t*_(14)_ = 1.39, *p* = 0.196. Note that our analysis showed no significant interaction between the factors emotion, orientation and hemisphere; *F*_(1, 14)_ = 2.49, *p* = 0.137, *η*^2^ = 0.151.

**Table 1:** **This table shows the means (standard deviations) of ERPs in microvolt for happy upright, fearful upright, happy inverted, and fearful inverted body expressions at occipital and frontal and central electrodes**.

		**Early visual processing N290:250–350 ms**	**Attention allocation Nc:700–800 ms**	
		**Occipital**	**Frontal and Central**	
		**O1/O2**	**F3/C3**	**F4/C4**
**happy upright**	Mean (SD) µV	6.42 (13.72)*	−3.08 (11.70)	−0.14 (10.78)*
**fearful upright**	Mean (SD) µV	−2.23 (11.79)*	−8.858 (9.02)	−9.19 (10.24)*
**happy inverted**	Mean (SD) µV	5.86 (17.06)	−2.46 (6.52)	−0.55 (6.24)
**fearful inverted**	Mean (SD) µV	−0.94 (14.35)	−7.65 (12.58)	−6.77 (11.10)

*p < 0.05.

*Nc*. Our analysis revealed a significant main effect of emotion at frontal and central electrodes from 700 to 800 ms, *F*_(1, 14)_ = 5.03, *p* = 0.042, *η*^2^ = 0.264, where fearful body expressions (*M* = −8.12 μV, *SE* = 2.15) elicited a more negative Nc than happy body expressions (*M* = −1.56 μV, *SE* = 1.79). Further analysis showed that this effect of emotion on the Nc was driven by differences over the right hemisphere for the upright orientation. Specifically, at the right-hemisphere fronto-central electrodes ERPs to upright fearful body expressions were significantly more negative in their mean amplitude (*M* = −9.19 μV, *SE* = 2.64) than ERPs elicited by upright happy expressions (*M* = −0.14 μV, *SE* = 2.78),* t*_(14)_ = 2.79, *p* = 0.014 (uncorrected; see Figure 1B and Table 1), whereas no significant differences were observed at the right-hemisphere fronto-central electrodes when the stimuli were inverted, *t*_(14)_ = 1.53, *p* = 0.147, or at the left-hemisphere fronto-central electrodes when the stimuli were presented upright, *t*_(14)_ = 1.52, *p* = 0.150 or inverted, *t*_(14)_ = 1.43, *p* = 0.174. Note that our analysis showed no significant interaction between the factors emotion, orientation and hemisphere, *F*_(1, 14)_ = 0.389, *p* = 0.543, *η*^2^ = 0.027.

Note that reported *p*-values for pairwise-comparisons are uncorrected. The *p*-value for the Nc survives a Bonferroni correction but for the N290 the *p*-value does not survive a Bonferroni correction threshold at *p* < 0.025.

## Discussion

The current study examined how infants process emotional information from body postures by investigating the neural correlates of discriminating between fearful and happy body expressions. Our results revealed two emotion-sensitive ERP responses (N290 and Nc) distinct in timing and topography. Namely, we found that 8-month-old infants discriminated between emotions as reflected in ERP differences for (a) the N290 at occipital electrodes during an early time window (250–350 ms); and (b) the Nc at frontal and central electrodes during a late time window (700–800 ms).

More specifically, the pattern of ERP findings indicates that this ability relies on early visual processes (N290, Kobiella et al., 2008) as revealed by the ERP difference observed at occipital electrodes and later attentional processes (Nc, Nelson and de Haan, 1996; Peltola et al., 2009) as indexed by the ERP difference observed at frontal and central electrodes. The early ERP effect on the N290, with an enhanced N290 elicited by fearful body expressions when compared to happy body expressions, is in line with prior work showing that the N290 varies as a function of emotional facial expressions (Kobiella et al., 2008). This suggests that emotional information affects early visual (posterior) processing likely related to the structural encoding of both bodies and faces (Halit et al., 2004; Gliga and Dehaene-Lambertz, 2005). Critically, the early occipital ERP effect appears to be specific to the discrimination processes elicited by static emotional body expressions, because it was only observed in the current study but not in prior ERP work using emotional PLDs (Missana et al., 2014a). This might have to do with the fact that in the current study the ERP response was measured in response to discrete emotional body postures (taken at the apex of the expression) enabling fast detection of differences in expression, while for the dynamic stimuli changes in body posture unfold more slowly over time and might thus be harder to detect for the infants. The later ERP effect on the Nc, with an enhanced Nc elicited by fearful body expressions when compared to happy body expressions, is in general agreement with prior work showing a similar effect on the Nc in response to fearful and happy facial expressions (Nelson and de Haan, 1996; Peltola et al., 2009). Interestingly, the enhanced Nc response to fearful expressions in infants is similar to a fronto-central response observed in prior work with adults (Stekelenburg and de Gelder, 2004), suggesting that both infants and adults possess neural processes associated with increased allocation of attention to fearful bodies. This speaks to the importance of fear signals in directing attention (Vuilleumier, 2005). The results indicate that by the age of 8-months the infant brain distinguishes between bodily expressions of emotion, even in static displays, which is consistent with previous research showing similar results in topography in adults’ brains. However, further research is required to directly compare and contrast the timing and topography of these responses (and ultimately, of the underlying neural processing) across infants and adults.

With respect to this finding concerning the Nc response it is important to note that the ERP difference observed for body expressions occurred somewhat later than the ERP difference commonly reported for facial expressions, suggesting that it might take infants longer to extract emotional information from bodies than from faces. Why this might be the case should be examined in future work that directly compares emotion processing from faces and bodies. Irrespective of these timing differences, the current data on body expression processing and prior work on facial expressions processing suggest that the detection of emotional information affects later anterior processing related to differential attention allocation to bodies and faces. Furthermore, this finding indicates that fearful expressions regardless of whether they are presented in the face or through body posture evoke a greater allocation of attention as indexed by the Nc. That the perception of signals of fear in others would result in such an effect possibly serves a critical adaptive function because it may allow infants from early in life to pay attention and learn from others in dangerous and threatening situations.

It is important to mention that, although not obtaining an interaction between orientation (upright, inverted) and emotion (fearful, happy), detailed analyses of the N290 and the Nc revealed that ERPs differed between emotions, specifically in the upright condition. This suggests that emotion effects were mainly driven by body posture seen in an upright orientation. This is similar to what is known from behavioral and ERP studies with adults (Stekelenburg and de Gelder, 2004; Atkinson et al., 2007) and infants (Missana et al., 2014a; Zieber et al., 2014) regarding emotional perception from body expressions. Critically, the disruption of emotion discrimination by body inversion can be seen as evidence for configural processing of body posture. That is, rather than relying on individual features of the body that are also present in the inverted stimulus, 8-month-olds require to see the familiar configuration of body features in order for the emotion discrimination process to take place. In this context, it is important to emphasize that this is one of the first infant ERP studies that investigated the effects of stimulus inversion on visual emotion processing (see Missana et al. (2014a), for the only other ERP study that used this kind of manipulation). Clearly, more work is needed to further specify the exact nature of orientation effects on visual emotion processing. However, it should be noted that using inverted stimuli as control stimuli yields an advantage over studies that used a neutral condition or no control condition because the low level visual information is kept identical across orientations, whereas neutral conditions generally differ with respect to low level visual features from the emotional conditions. Thus, the use of inverted stimuli can be seen as a strength of the current study.

Moreover, although we did not obtain an interaction between the factors hemisphere (left, right) and emotion (fearful, happy), we observed that for the Nc ERPs differed between fearful and happy body expressions only over the right hemisphere. This is similar to what has been shown in prior work using dynamic body expressions (Missana et al., 2014a), indicating that similar lateralization of the brain response can be observed for dynamic and static stimuli. In line with prior adult work (Heberlein et al., 2004; Heberlein and Saxe, 2005; Grèzes et al., 2007), this may suggest that the right hemisphere begins to play an important role in emotional body expression processing from early in ontogeny. Taken together, these results provide corroborating evidence for the laterality and orientation specificity of the brain processes employed by infants at the age of 8 months when perceiving emotional body expressions.

However, it should also be noted that the timing and the topography of the emotion-sensitive ERP effects varied between prior work using dynamic stimuli (Missana et al., 2014a) and the current study. These dissimilarities provide insights into how the processing of dynamic and static body expressions differs. As discussed above, the N290 effect appears to be unique to static presentation of emotional bodies. Furthermore, the current data showed a modulation for the Nc at frontal and central electrodes, whereas prior work using dynamic stimuli elicited later effects on the Pc at temporal and parietal electrodes. This suggests brain processes distinct in timing and region are engaged when discriminating between emotional body expressions on the basis of motion cues (Missana et al., 2014a) and posture cues (current study). Importantly, that dynamic and static presentation of emotional expressions evokes distinct processes in the human brain has been repeatedly shown in adults (Sato et al., 2004; Kessler et al., 2011), suggesting that it is of critical importance to study the emergence of these differences in development. This is a topic that has been greatly neglected as far as the neuroscience of emotion perception in infancy is concerned. Clearly, future work is needed that directly compares emotion processing from static and dynamic body cues within the same infants in order to better describe and understand the nature of these differences.

As mentioned in the methods section, we took several precautionary measures to control that the parents did not influence infants’ responses to the stimuli. We asked the parents not to attend to the screen, not to talk or interact with their infant during the course of the experiment. We further video-recorded the sessions so that trials during which the parent interfered with the procedure could be excluded from the analysis. Despite of all of these measures taken, we still cannot completely rule out the possibility that parents unintentionally cued the infants. This limitation applies to all infant studies in which the infants sit on their parents’ lap and might be addressed in future studies by placing the infant in a seat that prevents direct contact between the infant and the parent (e.g., Fairhurst et al., 2014).

In summary, the current findings have informed three main aspects of our understanding of how emotional body expressions are processed. First, as far as the developmental perspective is concerned, we have seen that 8-month-old infants detect emotional information from body postures, providing evidence that at this age they sensitively respond to emotional information from bodies. This sensitivity to emotional body expressions is manifested at a time in development when a similar sensitivity to facial and vocal emotional cues has developed (Peltola et al., 2009; Grossmann, 2013). In conjunction with prior work, these findings thus provide evidence for accounts that conceive of emotion perception as a unified ability that is reflected across various processing channels (face, voice and body). Second, with respect to the neurodynamics of body expression processing, we have seen that emotional body posture discrimination is reflected in early perceptual (visual) and later attentional neural processes, suggesting that emotion discrimination is multifaceted and relies on perceptual processes that occur before differential attention is allocated to emotional stimuli. Third, at the hemispheric level, we have provided evidence that emotion discrimination from body expressions elicits brain responses (Nc) that are more prominent in the right hemisphere. In agreement with prior work (Heberlein et al., 2004; Heberlein and Saxe, 2005; Grèzes et al., 2007), this suggests that the right hemisphere begins to play an important role in emotional body expression processing from early in ontogeny. All in all, the current data has shed new light on emotional body expression processing in infancy thereby critically extending and informing accounts of emotion understanding.

## Conflict of interest statement

The authors declare that the research was conducted in the absence of any commercial or financial relationships that could be construed as a potential conflict of interest.
